# Hall on Spinal Irritation

**Published:** 1853-08

**Authors:** G. W. Hall

**Affiliations:** Carthage, Ill.


### *A case of Spinal Irritation resulting in structural lesion.*

BY DR. G. W. HALL, OF CARTHAGE, ILL.

I was called on the 8th of January last, to visit Mrs. ———, æt 53. I learned that she had been a sufferer for six or eight years from various neuralgic pains, want of appetite, costiveness, accompanied with irregular and sometimes violent action of the heart, general debility and "nervousness."

About the first of November she was attacked with a severe pain in the left shoulder and arm, extending down the forearm to the fingers.

To relieve the intense pain she had used a variety of applications, and, she had consulted several physicians, been subject to several kinds of treatment—all of which only gave, at times, temporary relief. I found a great degree of tenderness over the lower cervical and upper dorsal vertebræ—firm pressure increasing the pain in the shoulder and arm, and causing a peculiar tingling sensation in the fingers.

By the recommendation of Dr. Judd, of Warsaw, she had been blistered and cupped over the affected spine, but had abondoned it with only a slight benefit. I gained her consent to again submit to a similar treatment, and this was rigidly adhered to for nearly two months, assisted by the use of laxatives, opiates, mineral acids and large doses of precipitated carb. of iron. At the end of two months she was no better, even worse; and so great was her distress that nothing but inordinate doses of opium would give her any ease, or produce sleep.

Belladonna and cicuta, and almost all the class of narcotics were repeatedly tried, and produced no beneficial effect. By my request Prof. McGugin was called in consultation.

Dr. McG. agreeing in the diagnosis, but a slight modification in the treatment was made, with but little hope. She gradually grew worse till the 10th of April, when she expired,—life, fraught with so many pleasures to the young and healthy, having been to her for years truly a burthen.

Autopsy.—Owing to the late hour when the consent of the friends was given, only a hurried examination could be made. The spinal marrow was examined from the 3d cervical to the 4th dorsal vertebræ. The meninges of the medalla spinalis were in a high state of inflammation—the spinal marrow completely softened, and the canal almost filled with effused serum. The body of the 3d dorsal vertebrœ was completely softened.

The time for investigation being limited, we were forced to cease the *post mortem* examination of this very interesting case.

I was assisted in the autopsy by my friend and former partner, Dr. Barnes, of this place.

Remarks.—Imperfect and hurried as the examination was, it enabled us to prove the correctness of our diagnosis, and demonstrate that any course of treatment would have been futile. This lady had been troubled for about three years before the final cessation of the menses, (which occurred at 45,) with troublesome and persistent drismenorrhœa. No doubt the spinal disease was a consequence of the previously existing uterine disturbance, and, in the language of Prof. McGugin, it is "an interesting case, as it shows how an irritation existing in a remote organ—as the uterus—may be transmitted to the spine, and be so obscured by a variety of symptoms as not to be suspected until its effects are irremediable;" and no doubt her indisposition for several years past was entirely owing to the latent and insidious, but generally extensive and fatal inflammation of the medulla spinalis and its meninges.

Imperfect as this account is, if by it others may be enabled to form a correct diagnosis in similar cases, and properly direct the treatment, *while treatment may be servicable*, the object for which it was reported will be fully attained.

